# Habitual Meat Consumption and Changes in Sleep Duration and Quality in Older Adults

**DOI:** 10.14336/AD.2018.0503

**Published:** 2019-04-01

**Authors:** Alberto Lana, Ellen A. Struijk, Lucía Arias-Fernandez, Auxiliadora Graciani, Arthur E. Mesas, Fernando Rodriguez-Artalejo, Esther Lopez-Garcia

**Affiliations:** ^1^Department of Medicine, Preventive Medicine and Public Health Area, School of Medicine and Health Sciences, Universidad de Oviedo, Spain.; ^2^Department of Preventive Medicine and Public Health, School of Medicine, Universidad Autónoma de Madrid, Spain /IdiPAZ, CIBER of Epidemiology and Public Health (CIBERESP), Madrid, Spain.; ^3^IMDEA-Food Institute, CEI UAM+CSIC, Madrid, Spain.; ^4^Department of Public Health, Universidade Estadual de Londrina, Londrina, PR, Brazil.

**Keywords:** meat, diet, sleep, aging, cohort study

## Abstract

Dietary proteins are sources of some amino acid precursors of two neurotransmitters relevant for biological rhythms, serotonin and melatonin, which are involved in sleep and alertness. Meat is the main source of proteins in many countries. Furthermore, meat consumption is of special interest because it provides high-quality protein as well as saturated and *trans* fatty acids. However, its effect on sleep patterns is unclear. Thereby, the aim was to examine the association of habitual meat consumption with changes in sleep duration and with sleep quality in older adults. We used data from 1,341 participants in the Seniors-ENRICA cohort aged ≥60 years, followed from 2012 through 2015. Habitual meat consumption was assessed at baseline with a validated diet history. Sleep duration and quality were ascertained both in 2012 and 2015. Analyses were performed with logistic regression and adjusted for socio-demographic variables, lifestyle, morbidity, sleep duration and poor sleep indicators at baseline. During follow-up, 9.0% of individuals increased and 7.9% decreased their sleep duration by ≥2 hours/night. Compared with individuals in the lowest tertile of meat consumption (<87 g/d), those in the highest tertile (≥128 g/d) showed increased incidence of a large decrease (≥2 h) in sleep duration (OR: 1.93; 95% CI:1.01-3.72; p-trend:0.04). Higher consumption of meat was also associated with incidence of snoring (OR:2.06; 95% CI:1.17-3.60; p-trend:0.01) and poor general sleep quality (OR:1.71; 95% CI:1.04-2.82; p-trend:0.03). Each 100 g/d increment in meat intake was associated with a 60% higher risk of both large sleep duration changes and poor sleep quality (OR:1.60; 95% CI:1.07-2.40). Results were in the same direction for red and processed meat and for white meat separately, and among individuals with physical impairment. Higher meat consumption (≥128 g/d) was associated with changes in sleep duration and with poor sleep in older adults.

Sleep disorders have been associated with higher risk of adverse health outcomes in older people. Several studies have found that altered sleep duration or quality are related to cardiometabolic diseases (1-3), cognitive decline (4,5) and the frailty syndrome (6,7), which suggests that sleep disorders can exacerbate the effects of the aging process (8). Therefore, keeping or achieving a good sleep pattern seems to be of central importance to maintain physical and mental function in the older age.

Diet is one of the lifestyle factors that has been linked to sleep patterns (9). For instance, higher adherence to a Mediterranean diet pattern may help to maintain stable sleep duration and to improve sleep quality (10). However, in short-term clinical trials diets with high carbohydrate intake impaired sleep quality (11,12). Also, lower fiber and higher fat intake, especially during the evening period, have been associated with lower sleep efficiency and quality (13,14).

The effect of dietary proteins on sleep is uncertain. Only a limited number of short-term randomized trials among young or middle-aged adults have explored whether high-protein diets (forced intakes ranging 1,5 to 2,4 g/kg/d, that is, between double and triple of the recommended dietary allowance) are related to sleep changes, and their results are inconclusive (15-17). In addition, these studies did not allow for understanding the effect of habitual protein intake on sleep patterns over the long term. Also, given that older adults should have a relatively high intake of protein (1.2-1.5 g/kg/d) to prevent sarcopenia and physical impairment (18), more evidence is needed on the effect of different protein sources on sleep on this fast-growing group of population. Meat is of special interest because it provides high-quality protein, but also saturated fatty acids and *trans* fatty acids that may be detrimental for health (19). Furthermore, meat is one of the main sources of proteins in the diet of many developed countries (20). However, to the date no previous study has specifically explored the role of meat consumption on sleep. Therefore, the aim of this study was to examine the prospective association of habitual meat consumption with changes in sleep duration and with sleep quality in community-dwelling older adults.

## MATERIALS AND METHODS

### Study design and participants

Data were taken from the Seniors-ENRICA cohort, whose methods have been reported elsewhere (21). Briefly, this cohort was established in 2008-2010 with 3,289 community dwelling people aged ≥60 years in Spain (wave I). Then, 2,519 individuals were followed until 2012 (wave II) and until 2015 (wave III). In all waves, data collection was performed using similar procedures. First, information on lifestyle, use of health services, health status and morbidity were collected through standardized phone interviews. Then, in two subsequent home visits, trained staff performed a physical examination and obtained a diet history. Given that sleep duration and quality were only measured in the last two waves, this study was a prospective analysis of the Seniors-ENRICA cohort between 2012 and 2015.

Written informed consent was given by all study participants prior to enrollment. The study was approved by the Clinical Research Ethics Committee of the *La Paz* University Hospital in Madrid.

## Study variables

### Meat consumption

At wave II, a validated computerized diet history, developed from that used in the EPIC-cohort study in Spain, was used to assess the habitual consumption of up to 880 foods during the previous year (22). This instrument included sets of photographs to help participants estimate the serving size. The diet history collected consumption of 54 types of meat, which were subsequently grouped into red and processed meat (beef, pork, Spanish cold cuts, sausages, hamburgers and others, including sheep, goat or horse), and white meat (chicken, rabbit and others, including turkey, quail or pheasant).

### Sleep duration and quality

Participants were asked to report sleep duration and quality in 2012 and 2015. First, hours (h) and minutes (m) of sleep duration were obtained with one single question about usual sleep pattern: ‘Approximately, for how long you usually sleep per night?’ Change in sleep duration was calculated by subtracting the time slept in 2015 from that in 2012. Then, changes in sleep duration were categorized as ‘no change’ (changes <30 min), ‘slight increase’ (increase ≥30 min and <2 h), ‘large increase’ (increase ≥2 h), ‘slight decrease’ (decrease ≥30 min and <2 h) and ‘large decrease’ (decrease ≥2 h) (2,10). These categories of sleep duration change were later dichotomized as ‘slight change’ (increase or decrease of ≥30 min and <2 h) and ‘large change’ (increase or decrease of ≥2 h), as both short and long sleep durations have been associated with adverse health outcomes (23,24).

Second, we estimated participants´ sleep quality with nine self-reported indicators. To obtain the first six indicators we measured the frequency of the following: difficulty falling asleep, awakening during the night, early awakening with difficulty of getting back to sleep, being so sleepy at daytime as to need a nap, not feeling rested in the morning and use of sleeping medications. These indicators of poor sleep were deemed to be present when participants rated them as ‘sometimes’ or ‘almost always’. Participants were also asked if they snored (yes/no) and about their general perception of sleep quality, which was categorized as ‘poor sleep’ (including ‘very poor’, ‘poor’ and ‘regular’) or ‘good sleep’ (‘good’ and ‘very good’). Finally, the Epworth Sleepiness Scale (ESS) was used to assess the level of daytime sleepiness (25). This validated questionnaire contains 8 different items, measured on a 0 to 3 scale, yielding a total score ranging from 0 to 24; a score >10 was considered as excessively sleepiness (25).

### Other variables

We also used data on potential confounders of the study association, including baseline socio-demographic characteristics and health behaviors. Specifically, we collected sex, age, education level (primary or less, secondary or university), physical activity at leisure time as assessed with the validated questionnaire from the EPIC study (26), smoking (never-, former-, current- smoker) and alcohol intake (g/d). Body mass index (BMI) was calculated as weight divided by squared height (kg/m^2^) and classified as <25.0, ≥25.0-29.9 and ≥30.0 kg/m^2^. Regarding diet, we calculated the Mediterranean Diet Adherence Screener (MEDAS) (27), which originally consists of 14 items with targets for food consumption and habits characteristic of this diet in Spain. A higher MEDAS score indicates better Mediterranean diet adherence and can be used as a proxy for a healthy diet pattern. Given that we preferred to independently adjust our analyses for alcohol intake and that the exposure variable was meat consumption, we excluded these two items from the MEDAS score. We also calculated caffeine (mg/d), sodium (g/d), cholesterol (g/d), saturated fatty acid (g/d) and total energy intake (Kcal) using standard Spanish food composition tables. Moreover, participants reported baseline and incident physician-diagnosed morbidity, including diabetes, cancer, cardiovascular and musculoskeletal diseases.

Additionally, we classified participants according to baseline physical impairment, including limitation in agility, mobility or to perform daily activities. Agility impairment was defined by answering "a lot" to the following question from the Rosow and Breslau Scale: "On a normal day, does your current health limit you to bend down or kneel?" (28). Mobility impairment was defined by answering “a lot” to any of the next three questions from the Rosow and Breslau scale: “On an average day with your current health, would you be limited in the following activities: 1) picking up or carrying a shopping bag?; 2) climbing one flight of stairs?; 3) walking several city blocks (a few hundred meters)?” (28). Lastly, limitation in activities of daily living was deemed to exist when participants scored ≥1 point in the Lawton and Brody's Scale (29) or ≤5 points in the Katz index (30).

**Figure 1.**
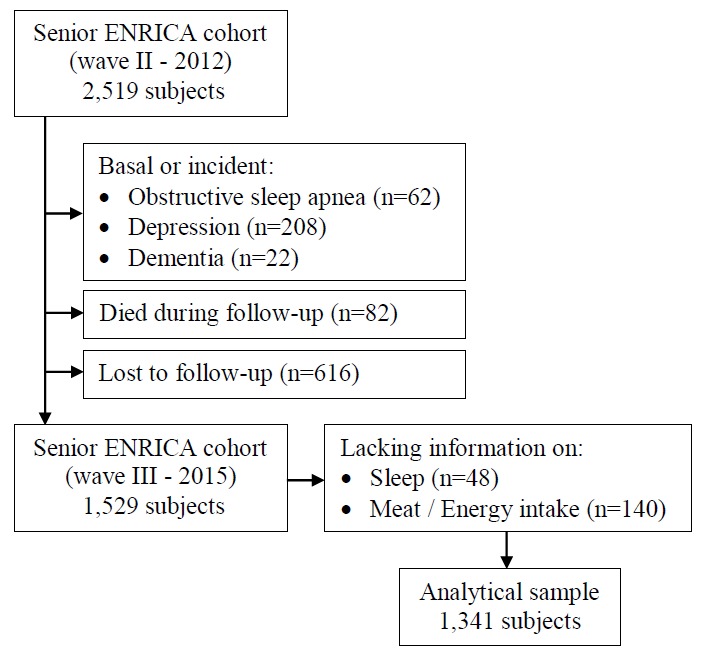
Study flow diagram.

**Table 1:** Baseline characteristics of the study participants according to meat consumption (n=1,341).

	Tertile 1 (<87 g/d)	Tertile 2	Tertile 3 (≥128 g/d)	p for trend
Participants, n	448	444	447	
Meat intake, g/d	62.0 (18.8)	106.3 (11.4)	173.8 (40.4)	
Age, y	69.0 (6.3)	67.7 (5.6)	67.2 (5.5)	<0.001
Gender, men, %	37.1	52.0	68.2	<0.001
Primary education, %	51.6	48.2	43.4	0.02
Leisure-time physical activity, MET-h/wk	22.5 (13.7)	22.5 (15.1)	24.4 (15.9)	0.99
Current smoker, %	15.2	6.2	11.9	0.04
Alcohol intake, g/d	7.2 (10.7)	8.9 (12.6)	13.5 (16.2)	<0.001
Caffeine intake, mg/d	79.4 (129.8)	93.8 (143.8)	100.6 (178.0)	<0.001
Sodium, g/d	2.2 (0.7)	2.6 (0.7)	3.3 (1.1)	<0.001
Cholesterol, g/d	246 (95)	295 (97)	360 (125)	<0.001
Saturated fatty acids intake, g/d	21.7 (8.1)	24.4 (8.4)	28.5 (10.1)	<0.001
Energy, kcal/d	1,855 (367)	2,006 (395)	2,274 (520)	<0.001
MEDAS score^a^	6.5 (1.6)	6.3 (1.5)	6.3 (1.6)	0.06
BMI, kg/m^2^	27.6 (15.9)	28.1 (19.1)	29.1 (18.4)	0.02
Morbidity, %				
Diabetes	13.6	21.3	20.3	0.86
Cancer	3.0	2.3	2.2	0.82
Cardiovascular disease	6.1	4.3	5.4	0.17
Osteomuscular disease	47.0	49.1	42.0	0.006
Sleep duration in 2012, h	6.8 (1.3)	7.0 (1.3)	6.9 (1.3)	0.66
Sleep duration in 2015, h	7.0 (1.3)	6.9 (1.3)	7.0 (1.4)	0.55
No changes in sleep duration from 2012 to 2015, %	36.4	36.0	34.4	0.31
Increase in sleep duration, min	77.2 (48.7)	72.9 (37.5)	80.8 (45.9)	0.42
Decrease in sleep duration, min	76.5 (47.8)	87.7 (52.0)	85.9 (54.4)	0.17
Indicators of poor sleep quality in 2012^b^, n	2.9 (1.8)	2.8 (1.7)	2.8 (1.7)	0.56
Indicators of poor sleep quality in 2015^b^, n	2.8 (1.7)	2.9 (1.7)	2.8 (1.6)	0.72

a Mediterranean Diet Adherence Screener (excluding meat and alcohol intake).

b Indicators of poor sleep quality are: poor general sleep quality, difficulty falling asleep, awakening during the night, early awakening with difficulty of getting back to sleep, need to sleep at daytime, not feeling rested in the morning, use of sleeping medication, snoring and daytime sleepiness (Epworth Sleepiness Scale >10).

BMI: Body mass index.

For continuous variables, means (standard deviations) are reported.

Socio-demographic, lifestyle and morbidity values are from 2012.

## Data analyses

Among the 2,519 initial participants, we excluded those with baseline (2012) or incident (2015) physician-diagnosed obstructive sleep apnea (n=62), depression (n=208) and dementia (n=22), because these chronic conditions are so strongly related to sleep disorders that is implausible that the consumption of a sole food group can modify their sleep duration or quality (31,32). Then, excluded 82 individuals who died during the follow-up and 616 who were lost. From the remaining 1,529 subjects, we excluded 48 with missing information on sleep variables and 138 on meat consumption. Additionally, we excluded 2 participants with an implausibly high or low energy intake (500-4000 kcal/d in women and 800-5000 kcal/d in men). Therefore, the analyses were conducted in 1,341 individuals (Fig. 1).

Participants were classified into tertiles of meat consumption and the lowest one was used as reference in the analyses: cut-off points were 87.09 and 127.78 g/d, but for readability, these figures are rounded up to 87 and 128 g/d in the text and tables. Multinomial logistic regression models were used to examine the association between tertiles of meat consumption and categories of sleep duration change from 2012 to 2015, using no change as the reference. In addition, we calculated the probability of changes in sleep duration associated with a 100 g/d increase in meat consumption. A first model was adjusted for sociodemographic variables (sex, age and educational level) and for baseline sleep duration; and a second model was additionally adjusted for health behaviors (physical activity, smoking, alcohol and caffeine intake, BMI, MEDAS score and energy intake) and for baseline and incident morbidity. In addition, estimates were adjusted for intake of sodium, dietary cholesterol and saturated fatty acids, to examine whether these nutrients might account for the studied association. Similar multinomial logistic regression models were used to examine the simultaneous effect of meat on both change in sleep duration and sleep quality; for this purpose, participants were classified in combined categories of large change in sleep duration (change <2 or ≥2 hours) and having ≥2 indicators of poor sleep at 2015. Adjusted odds ratios (OR) and their 95% confidence intervals (CI) were calculated for having 1 or 2 endpoints at follow-up, using 0 endpoint as reference. For this analysis, the models were additionally adjusted for the number of indicators of poor sleep at baseline.

**Table 2:** Odds ratios (95% confidence interval) for the association between tertiles of meat consumption and change in sleep duration during a 2.8 years follow-up of older adults (n=1,341).

	Tertile 1 (<87 g/d)	Tertile 2	Tertile 3 (≥128 g/d)	p for trend
No change^a^, n	163	160	148	
Reference category	1.00	1.00	1.00	
Increase ≥30 min to <2 h, n	133	112	133	
Model 1	1.00	0.90 (0.64-1.27)	1.20 (0.85-1.70)	0.29
Model 2	1.00	0.94 (0.66-1.33)	1.29 (0.90-1.86)	0.18
Model 3	1.00	0.96 (0.66-1.37)	1.40 (0.94-2.10)	0.10
Increase ≥2 h, n	37	35	49	
Model 1	1.00	1.07 (0.63-1.81)	1.76 (1.05-2.94)	0.03
Model 2	1.00	1.10 (0.64-1.91)	1.61 (0.92-2.80)	0.09
Model 3	1.00	1.18 (0.66-2.09)	1.76 (0.95-3.25)	0.07
Decrease ≥30 min to <2 h, n	90	91	84	
Model 1	1.00	1.10 (0.76-1.61)	1.25 (0.85-1.85)	0.27
Model 2	1.00	1.18 (0.80-1.75)	1.31 (0.86-1.99)	0.20
Model 3	1.00	1.20 (0.80-1.80)	1.42 (0.90-2.34)	0.13
Decrease ≥2 h, n	25	48	33	
Model 1	1.00	1.90 (1.07-3.37)	1.99 (1.07-3.69)	0.03
Model 2	1.00	1.90 (1.05-3.42)	1.93 (1.01-3.72)	0.04
Model 3	1.00	2.06 (1.11-3.85)	2.27 (1.10-4.66)	0.03

Slight change^b^ (<2 h), n	223	203	217	
Model 1	1.00	0.98 (0.73-1.31)	1.22 (0.90-1.66)	0.21
Model 2	1.00	1.03 (0.76-1.40)	1.29 (0.93-1.75)	0.13
Model 3	1.00	1.05 (0.77-1.46)	1.40 (0.99-1.99)	0.06
Large change^b^ (≥2 h), n	62	83	82	
Model 1	1.00	1.38 (0.91-2.09)	1.84 (1.20-2.82)	0.005
Model 2	1.00	1.36 (0.89-2.08)	1.68 (1.07-2.09)	0.02
Model 3	1.00	1.48 (0.95-2.31)	1.89 (1.15-3.10)	0.01

a Less than 30 minutes of change in sleep duration.

b Increase or decrease.

Model 1: multinomial logistic regression adjusted for sex, age (<70, 70-75, ≥75 y), educational level (≤ primary, secondary, university) and sleep duration in 2012 (<7, 7-9, >9 h).

Model 2: multinomial logistic regression additionally adjusted for physical activity (METs-h/wk, tertiles), smoking (never smoker, former smoker, current smoker), alcohol (g/d, tertiles), caffeine (mg/d, tertiles), BMI (<25, 25-29.9, ≥30 kg/m^2^), diet quality index (MEDAS score, tertiles), energy intake (kcal/d, tertiles) and baseline and incident morbidity, including diabetes, cancer, cardiovascular and osteomuscular diseases.

Model 3: multinomial logistic regression additionally adjusted for sodium (g/d, tertiles), cholesterol (g/d, tertiles) and saturated fatty acids intakes (g/d, tertiles).

To explore the association between tertiles of meat consumption and the incidence of each of the 9 sleep indicators separately, we used dichotomous logistic regression, with the same adjustment as above; for these analyses, we first eliminated prevalent cases of the 9 sleep indicators at baseline.

Finally, given that red and processed meat differ from white meat in the fat and vitamin content, we replicated the analyses separately for these two subtypes of meat. Also, because a high-protein diet can be recommended for older adults with physical limitations, we repeated the analyses only with subjects with baseline physical impairment (n=428). The linear dose-response relationship was tested with a p for linear trend by modeling tertiles of meat consumption as a continuous variable. A 2-tailed p value <0.05 was considered as statistically significant. Statistical analyses were performed with Stata, version 13.0 (Stata Corp., College Station).

**Table 3:** Odds ratios (95% confidence interval) for the association between tertiles of meat consumption and the incidence of each indicator of poor sleep quality during a 2.8 years follow-up of older adults (n=1,341).

	Tertile 1 (<87 g/d)	Tertile 2	Tertile 3 (≥128 g/d)	p for trend
Difficulty falling asleep, n cases/N	52/270	61/286	55/319	
Model 1	1.00	1.25 (0.82-1.91)	1.12 (0.72-1.72)	0.64
Model 2	1.00	1.22 (0.79-1.89)	1.07 (0.67-1.72)	0.78
Model 3	1.00	1.17 (0.74-1.84)	0.96 (0.58-1.60)	0.72
Awakening during the night, n cases/N	102/162	97/163	102/166	
Model 1	1.00	0.89 (0.56-1.40)	0.97 (0.61-1.55)	0.90
Model 2	1.00	0.85 (0.52-1.36)	0.99 (0.59-1.66)	0.96
Model 3	1.00	0.77 (0.47-1.29)	0.84 (0.48-1.47)	0.55
Early awakening, n cases/N	64/243	68/246	66/251	
Model 1	1.00	1.10 (0.73-1.65)	1.08 (0.71-1.64)	0.72
Model 2	1.00	1.12 (0.73-1.70)	1.06 (0.66-1.68)	0.81
Model 3	1.00	1.11 (0.72-1.73)	1.14 (0.69-1.89)	0.60
Need to sleep at daytime, n cases/N	52/391	31/378	44/378	
Model 1	1.00	0.57 (0.36-0.94)	0.86 (0.55-1.35)	0.48
Model 2	1.00	0.58 (0.35-0.95)	0.85 (0.53-1.37)	0.46
Model 3	1.00	0.57 (0.34-0.94)	0.77 (0.45-1.31)	0.31
Not feeling rested in the morning, n cases/N	48/353	36/360	30/359	
Model 1	1.00	0.78 (0.49-1.25)	0.71 (0.43-1.18)	0.17
Model 2	1.00	0.75 (0.46-1.24)	0.68 (0.39-1.17)	0.15
Model 3	1.00	0.79 (0.47-1.32)	0.75 (0.41-1.37)	0.33
Use of sleeping medications, n cases/N	27/347	42/335	32/347	
Model 1	1.00	1.79 (1.07-2.99)	1.42 (0.81-2.46)	0.22
Model 2	1.00	1.90 (1.10-3.27)	1.49 (0.81-2.72)	0.20
Model 3	1.00	2.09 (1.19-3.69)	1.55 (0.80-2.94)	0.19
Snoring, n cases/N	37/184	38/173	48/149	
Model 1	1.00	1.08 (0.64-1.82)	1.85 (1.11-3.09)	0.02
Model 2	1.00	1.18 (0.68-2.04)	2.06 (1.17-3.60)	0.01
Model 3	1.00	1.26 (0.71-2.25)	2.13 (1.14-3.99)	0.02
Poor general sleep quality, n cases/N	39/329	51/335	53/330	
Model 1	1.00	1.44 (0.92-2.27)	1.67 (1.05-2.66)	0.03
Model 2	1.00	1.38 (0.86-2.21)	1.71 (1.04-2.82)	0.03
Model 3	1.00	1.29 (0.79-2.12)	1.35 (0.78-2.33)	0.29
Daytime sleepiness^a^, n cases/N	49/382	38/374	37/369	
Model 1	1.00	0.80 (0.50-1.27)	0.86 (0.53-1.38)	0.50
Model 2	1.00	0.75 (0.46-1.21)	0.79 (0.47-1.31)	0.34
Model 3	1.00	0.70 (0.43-1.16)	0.69 (0.40-1.20)	0.18

a >10 points in the Epworth Sleepiness Scale. Analyses based on 1,240 participants who completed the questionnaire. Tertile cut-off points for meat were 87.1 and 127.8 g/d.

Model 1: logistic regression model adjusted for age, sex and educational level (≤ primary, secondary, university).

Model 2: logistic regression model additionally adjusted for physical activity (METs-h/wk, tertiles), smoking (never smoker, former smoker, current smoker), alcohol (tertiles of g/d), caffeine (mg/d, tertiles), BMI (<25, 25-29.9, ≥30 kg/m2), diet quality index (tertiles of MEDAS score), energy intake (kcal/d, tertiles) and baseline and incident morbidity, including diabetes, cancer, cardiovascular and musculoskeletal diseases.

Model 3: logistic regression additionally adjusted for sodium, cholesterol and saturated fatty acids intakes.

## RESULTS

At baseline, study participants consumed a mean (SD) of 114.0 (53.1) g/d of meat. Of this amount, 77.2 g/d was from red and processed meat and 36.8 g/d from white meat (Supplementary Table 1). Specifically, chicken was the most consumed type of meat (29.8 g/d), followed by beef (20.8 g/d) and Spanish cold cuts (19.6 g/d). In this population, meat provided 27.6% of total dietary protein.

Mean (SD) sleep duration was 6.9 (1.3) hours/night in 2012 and 7.0 (1.3) hours/night in 2015. Table 1 shows the baseline characteristics of the study participants according to tertiles of meat consumption. Compared with those in the lowest tertile, those with higher meat consumption were younger, more frequently men and with higher educational level. Also, they were more frequently smokers, with higher BMI, alcohol, caffeine, sodium, cholesterol, saturated fats and energy intake, and lower adherence to the Mediterranean diet. Moreover, they showed lower prevalence of osteomuscular disease.

Over a mean follow-up of 2.8 years, 9.0% of individuals increased and 7.9% decreased their sleep duration by ≥2 hours/night. In full adjusted analyses, compared to individuals in the lowest tertile of meat consumption, those in tertiles 2 and 3 showed higher incidence of a large decrease (≥2 h) in sleep duration (T2 OR: 1.90; 95% CI: 1.05-3.42; T3 OR: 1.93; 95% CI: 1.00-3.72; p-trend: 0.04) (Table 2). The ORs associated with a large change (increase or decrease) in sleep duration were 1.36 (95% CI: 0.89-2.08) and 1.68 (1.07-2.09), respectively; p-trend: 0.02). When we additionally adjusted the estimates for sodium, cholesterol and saturated fatty acid intakes, the results held the statistical significance.

Regarding sleep quality, being in the highest tertile of meat consumption was linked to higher risk of snoring (OR: 2.06; 95% CI: 1.17-3.60; p-trend: 0.01) and of poor general sleep quality (OR: 1.71; 95% CI: 1.04-2.82; p-trend: 0.03) (Table 3). Moreover, in the combined endpoints analysis, participants with the highest meat consumption had a higher risk of having both, a large change in duration and ≥2 indicators of poor sleep quality (OR: 1.96; 95% CI: 1.14-3.35; p-trend: 0.01) (Table 4). Each 100 g/d increase in meat consumption was associated with increased risk of developing 2 endpoints (OR: 1.60; 95% CI: 1.07-2.40). The magnitude of the association slightly decreased when the model was additionally adjusted for sodium, cholesterol and saturated fatty acids.

**Table 4:** Odds ratios (95% confidence interval) for the association between meat consumption and both change in sleep duration and number of indicators of poor sleep quality during a 2.8 years follow-up of older adults (n=1,341).

	Tertile 1 (<87 g/d)	Tertile 2	Tertile 3 (≥128 g/d)	p for trend	Continuous per 100 g/d increment
0 endpoint^a^, n	100	91	84		
Reference category	1.00	1.00	1.00		
1 endpoint, n	294	283	288		
Model 1	1.00	1.06 (0.75-1.52)	1.26 (0.87-1.81)	0.23	1.15 (0.86-1.54)
Model 2	1.00	1.01 (0.69-1.46)	1.26 (0.86-1.87)	0.29	1.17 (0.86-1.59)
Model 3	1.00	0.98 (0.66-1.43)	1.16 (0.76-1.78)	0.24	1.09 (0.77-1.53)
2 endpoints, n	54	72	75		
Model 1	1.00	1.43 (0.87-2.35)	2.08 (1.25-3.45)	0.004	1.68 (1.14-2.45)
Model 2	1.00	1.33 (0.80-2.22)	1.96 (1.14-3.35)	0.01	1.60 (1.07-2.40)
Model 3	1.00	1.35 (0.79-3.29)	1.78 (1.00-3.22)	0.05	1.44 (0.92-2.26)

a Endpoints are: large changes (increase or decrease) in sleep duration (≥2 hours) and ≥2 indicators of poor sleep quality

Model 1: multinomial logistic regression adjusted for sex, age (<70, 70-75, ≥75 y), educational level (≤ primary, secondary, university), sleep duration (<7, 7-9, >9 h) in 2012 and indicators of poor sleep in 2012 (≤1, 2-3, ≥4).

Model 2: multinomial logistic regression additionally adjusted for physical activity (METs-h/wk, tertiles), smoking (never smoker, former smoker, current smoker), alcohol (g/d, tertiles), caffeine (mg/d, tertiles), BMI (<25, 25-29.9, ≥30 kg/m^2^), diet quality index (MEDAS score, tertiles), energy intake (kcal/d, tertiles) and baseline and incident morbidity, including diabetes, cancer, cardiovascular and musculoskeletal diseases.

Model 3: multinomial logistic regression additionally adjusted for sodium (g/d, tertiles), cholesterol (g/d, tertiles) and saturated fatty acids intakes (g/d, tertiles).

Results were in the same direction but of less magnitude when analyses were repeated separately for red and processed meat and for white meat (data not shown). Finally, among adults with physical function impairment, higher meat intake increased the frequency of both slight and large modifications (either increase or decrease) in sleep duration (Supplementary Table 2). Results for sleep quality indicators (Supplementary Table 3) and for the combined endpoints (Supplementary Table 4) were similar to those in the total sample. All associations between meat consumption and sleep variables were of higher magnitude on the subsample of physically impaired participants than in the whole sample.

## DISCUSSION

In this study among older adults, higher habitual meat consumption was associated with large changes in sleep duration and with poor sleep quality, and both long decrease and increase in sleep duration have potential to trigger adverse physiological changes. Since the relationship between sleep quantity and quality and adverse health outcomes in older people has been extensively shown (1-7), our results are of relevance because they suggest that reducing meat intake may improve sleep patterns.

So far, the only study that can be used to compare our results was a randomized trial that examined the effect of fish versus meat consumption on sleep (33). In this study, the experimental group received 300 g of fatty fish 3 times/week for 6 months and the control group the same amount of meat. Sleep parameters were objectively measured using actigraphy during one week before the start and at the end of the intervention. They found a beneficial impact of fish on sleep, based on a pre-post comparison between groups. Given that there also was an increase of sleep latency and of wake time and a decrease of sleep efficiency in the control group, this suggests a detrimental effect of meat on sleep, which is consistent with our results. Nevertheless, their results referred to meat should be interpreted with prudence, as their study was not specifically designed to drawn conclusion about the effects of its consumption on sleep patterns.

Other studies have attempted to ascertain the effect of protein intake on sleep duration and quality (15-17,34,35). However, despite they used relatively comparable methodological approaches, it is not easy to draw a conclusion from them because of their conflicting results. For instance, two very similar experimental studies in overweight subjects during a period of weight loss, reported contradictory results: whilst Karl *et al* found no effect of high-protein intake on subjective sleep quality (17), Zhou *et al* reported that proteins may be beneficial (16). In another well designed experimental study, using a crossover design and actigraphy monitors to measure sleep variables, subjects under a very high-protein diet showed significantly fewer nocturnal wake episodes (15). In any case, these trials were conducted with young or middle-aged adults, who usually have less sleep disorders and different physiological response to nutrients than older adults, and assessed short-term effects of diet. Moreover, and despite the risk of reverse causality, a few cross-sectional population-based studies have also tried to assess the association between protein and sleep (34,35). The secondary analyses of the United States National Health and Nutrition Examination Survey carried out by Grandner *et al* show that an increased protein intake was associated with a decreased likelihood of very short sleep (34), and Liu *et al* using data form the Chinese Health and Nutrition Survey, found an age-interaction: protein intake was associated with less sleep duration but only among older adults (35).

Given that results were in the same direction for red and processed meat than for white meat and that there were only little changes in the results after adjusting for nutrients that might have a detrimental effect, the main harmful effect of meat on sleep might be exerted through their protein content. The most prominent mechanism of the effect of high-protein diets on sleep could be related to two amino acids: tryptophan (TRP) and tyrosine (TYR) and their capacity to synthesize melatonin, serotonin and dopamine, which are involved in the sleep-wake cycle (36). Ratios between both circulating TRP and TYR and large neutral amino acids (TRP: LNAA and TYR:LNAA) regulate brain levels of TRP and TYR, which are precursors of melatonin, serotonin and dopamine (37,38). It was previously suggested that high-protein intake reduced these ratios and therefore, lead to a reduction in the synthesis of brain sleep inductors and to a deterioration of sleep parameters, which is in line with our results (37).

Moreover, some age-related characteristics could explain the stronger effect of protein intake on sleep among older people, specially in those with physical impairment. Reduced insulin sensitivity and sarcopenia, which are common age-related disorders (39), could contribute to a slowdown in the uptake of circulating LNAA into muscles, which would lead to a lower blood TRP: LNAA ratio and to lower brain levels of TRP (40). Furthermore, two population-based studies found that consumption of red and processed meat has been associated with circulating inflammation markers (41,42), including C-reactive protein and interleukin-6, which have been linked to sleep disorders (1). In these studies, mean intake of red and processed meat were 65-110 g/d among men and 50-57 g/d among women. Given that consumption of red and processed meats in our cohort was the most consumed group of meat and mean intakes were within those ranges, this mechanism might explain our results.

Lastly, in our study high meat consumption was independently associated with incident snoring and poor general sleep quality. In fact, we cannot ensure whether these sleep indicators are actually pointing to incident obstructive sleep apnea, since the occurrence of this syndrome is common during aging (43). However, in previous studies, neither protein intake nor fat intake seemed to be independent risk factors for obstructive sleep apnea (44). Thereby, more research is probably needed to elucidate the role of meat consumption over these sleep indicators. In any case, both obstructive sleep apnea syndrome and snoring alone are related with relevant adverse health conditions (43,45).

This study has some limitations. The main one is related to the subjective measurements of sleep, with the potential for information bias. We were not able to objectively measured sleep in this cohort. Many population studies have also relied on self-reported sleep duration since the complexity to use polysomnography or actigraphy in large free-living populations (35,46,47). In addition, self-reported sleep duration has been included in the Pittsburgh Sleep Quality Index (48-50), which is a validated and well-established questionnaire to assess sleep quality. Although some studies assessed sleep timing separately for weekdays and weekends (51), we believed that this is not relevant for our population of older adults, who were mostly retired. Second, despite we used a validated diet history, there could be some recall bias in diet assessment. Nevertheless, this type of bias tends to underestimate study associations. Third, given that the consumption of red and processed meat jointly included lean and fat meat, whether the effect on sleep is exerted by fat or protein content cannot be completely known. However, it is more plausible that the effect was due to proteins, since the analyses about white meat were in the same direction. Fourth, reverse causation cannot be completely ruled out; longer follow-up periods are required to confirm our results in population-based analyses using habitual diet. Lastly, despite the analyses were adjusted for a good number of variables, some residual confounding may persist.

## Conclusion

In conclusion, in our cohort of older adults, high habitual meat consumption (particularly ≥128 g/d) was prospectively associated with poorer sleep patterns. The magnitude of the association was higher among those with physical function impairment. Given that a relatively high protein intake should be ensured in the older population to prevent frailty and sarcopenia, further research must identify types of food with an optimal balance between the content of high-quality protein and other nutrients with positive effects on sleep patterns, so that the potential detrimental effect of protein might be compensated. Furthermore, in order to provide more consistency to our findings, these researches should ideally include some objective measure of sleep duration and quality.

## Supplementary Materials

The Supplemenantry data can be found online at: www.aginganddisease.org/EN/10.14336/AD.2018.0503
