# Supplementary material for: Habitual Meat Consumption and Changes in Sleep Duration and Quality in Older Adults
Source: Aging Dis. 2019 Apr 1;10(2):267–77. doi: 10.14336/AD.2018.0503 (PMC6457059; doi:10.14336/AD.2018.0503)
Supplement: Supplementary file 1 — The Supplemenantry data can be found online at: www.aginganddisease.org/EN/10.14336/AD.2018.0503 [file AD-10-2-267-s.pdf]

SUPPLEMENTARY DATA

Habitual Meat Consumption and Changes in Sleep Duration and Quality in Older Adults

Alberto Lana[1,2, *], Ellen A. Struijk[2], Lucía Arias-Fernandez[1,2], Auxiliadora Graciani[2], Arthur E. Mesas[4], Fernando Rodriguez-Artalejo[2,3], Esther Lopez-Garcia[2,3, *]

[1]Department of Medicine, Preventive Medicine and Public Health Area, School of Medicine and Health Sciences, Universidad de Oviedo, Spain.
[2]Department of Preventive Medicine and Public Health, School of Medicine, Universidad Autónoma de Madrid, Spain /IdiPAZ, CIBER of Epidemiology and Public Health (CIBERESP), Madrid, Spain.
[3]IMDEA-Food Institute, CEI UAM+CSIC, Madrid, Spain.
[4]Department of Public Health, Universidade Estadual de Londrina, Londrina, PR, Brazil.

© 2018. Lana A et al. Published online at http://www.aginganddisease.org/EN/10.14336/AD.2018.0503

SUPPLEMENTARY DATA

Supplementary Table 1. Mean (standard deviation) of baseline consumption of each subtype of meat.

Overall meat, g/d	114.0 (53.1)
Red and processed meat, g/d	77.2 (46.4)
Beef, g/d	20.8 (24.3)
Spanish cold cut, g/d	19.6 (22.2)
Processed, g/d	17.0 (25.5)
Pork, g/d	8.8 (10.6)
Other, g/d	11.0 (15.3)
White meat, g/d	36.8 (28.8)
Chicken, g/d	29.8 (24.6)
Rabbit, g/d	3.2 (7.5)
Other, g/d	3.8 (10.2)

Supplementary Table 2 Odds ratios (95% confidence interval) for the association between tertiles of meat consumption and change in sleep duration during a 2.8 years follow-up of older adults with some physical impairment[a] (n= 428).

	Tertile 1 (<87 g/d)	Tertile 2	Tertile 3 (≥128 g/d)	p for trend
No changeb, n	60	39	25	
Reference category	1.00	1.00	1.00	
Increase ≥30 min to <2 h, n	40	35	35	
Model 1	1.00	1.46 (0.77-2.76)	2.50 (1.25-5.02)	0.01
Model 2	1.00	1.76 (0.87-3.53)	3.89 (1.74-8.67)	0.001
Model 3	1.00	1.81 (0.86-3.77)	3.37 (1.40-8.14)	0.007
Increase ≥2 h, n	16	12	25	
Model 1	1.00	1.43 (0.58-3.53)	4.32 (1.84-10.21)	0.001
Model 2	1.00	1.82 (0.66-5.04)	6.17 (2.17-17.70)	0.001
Model 3	1.00	2.22 (0.76-6.44)	7.07 (2.13-23.45)	0.001
Decrease ≥30 min to <2 h, n	38	31	23	
Model 1	1.00	1.10 (0.57-2.15)	1.90 (0.90-4.00)	0.11
Model 2	1.00	1.26 (0.61-2.58)	2.31 (1.00-5.41)	0.05
Model 3	1.00	1.15 (0.55-2.44)	1.88 (0.84-4.81)	0.12
Decrease ≥2 h, n	11	22	16	
Model 1	1.00	2.11 (054-5.32)	4.52 (1.65-12.42)	0.004
Model 2	1.00	2.02 (0.72-5.68)	3.71 (1.17-11.79)	0.03
Model 3	1.00	2.10 (0.68-6.46)	3.28 (1.01-12.84)	0.08
Slight changec (<2 h), n	78	66	58	
Model 1	1.00	1.27 (0.74-2.21)	2.21 (1.19-4.09)	0.01
Model 2	1.00	1.50 (0.83-2.72)	3.01 (1.49-6.04)	0.002
Model 3	1.00	1.51 (0.82-2.81)	2.61 (1.22-5.63)	0.01
Large changec (≥2 h), n	27	34	41	
Model 1	1.00	1.69 (0.84-3.99)	4.42 (2.12-9.21)	<0.001
Model 2	1.00	1.77 (0.83-3.76)	4.64 (2.01-10.71)	<0.001
Model 3	1.00	2.03 (0.93-4.43)	5.50 (2.17-12.97)	<0.001

[a] Basal impaired mobility, impaired agility, Katz index≤5 or Lawton-Brody index≥1.
[b] Less than 30 minutes of change in sleep duration.
[c] Increase or decrease.
Model 1: multinomial logistic regression adjusted for sex, age (<70, 70-75, ≥75 y), educational level (≤ primary, secondary, university) and sleep duration in 2012 (<7, 7-9, >9 h).
Model 2: multinomial logistic regression additionally adjusted for physical activity (METs-h/wk, tertiles), smoking (never smoker, former smoker, current smoker), alcohol (g/d, tertiles), caffeine (mg/d, tertiles), BMI (<25, 25-29.9, ≥30 kg/m2), diet quality index (MEDAS score, tertiles), energy intake (kcal/d, tertiles) and baseline and incident morbidity, including diabetes, cancer, cardiovascular and musculoskeletal diseases.
Model 3: multinomial logistic regression additionally adjusted for sodium (g/d, tertiles), cholesterol (g/d, tertiles) and saturated fatty acids intakes (g/d, tertiles).

© 2018. Lana A et al. Published online at http://www.aginganddisease.org/EN/10.14336/AD.2018.0503

Supplementary Table 3. Odds ratios (95% confidence interval) for the association between tertiles of meat consumption and the incidence of each indicator of poor sleep quality during a 2.8 years follow-up of older adults with some physical impairment[a] (n= 428).

	**Tertile 1 (<87 g/d)**	**Tertile 2**	**Tertile 3 (≥128 g/d)**	**p for trend**
Difficulty falling asleep, n cases/N	17/78	19/68	20/74	
Model 1	1.00	1.47 (0.68-3.18)	1.73 (0.79-3.79)	0.17
Model 2	1.00	1.48 (0.60-3.65)	2.65 (1.01-6.91)	0.04
Model 3	1.00	1.44 (0.59-3.55)	2.29 (0.90-5.58)	0.08
Awakening during the night, n cases/N	33/46	23/38	27/37	
Model 1	1.00	0.65 (0.24-1.71)	1.18 (0.41-3.42)	0.83
Model 2	1.00	0.37 (0.10-1.58)	0.65 (0.10-4.09)	0.40
Model 3	1.00	0.19 (0.03-1.32)	0.32 (0.04-3.15)	0.17
Early awakening, n cases/N	27/75	17/61	26/65	
Model 1	1.00	0.67 (0.32-1.41)	1.30 (0.62-2.71)	0.55
Model 2	1.00	0.68 (0.31-1.55)	1.62 (0.65-4.03)	0.44
Model 3	1.00	0.54 (0.22-1.31)	1.89 (0.67-5.34)	0.45
Need to sleep at daytime, n cases/N	15/135	14/117	14/96	
Model 1	1.00	1.06 (0.47-2.36)	1.28 (0.57-2.89)	0.56
Model 2	1.00	0.94 (0.38-2.28)	1.44 (0.55-3.71)	0.50
Model 3	1.00	0.90 (0.36-2.24)	1.42 (0.48-4.22)	0.59
Not feeling rested in the morning, n cases/N	24/119	14/100	12/88	
Model 1	1.00	0.70 (0.33-1.46)	0.84 (0.38-1.86)	0.57
Model 2	1.00	0.62 (0.27-1.43)	0.91 (0.35-2.36)	0.69
Model 3	1.00	0.72 (0.30-1.71)	1.23 (0.41-3.63)	0.88
Use of sleeping medications, n cases/N	17/114	14/99	12/80	
Model 1	1.00	1.15 (0.52-2.55)	1.07 (0.47-2.46)	0.85
Model 2	1.00	1.02 (0.42-2.45)	0.90 (0.33-2.43)	0.78
Model 3	1.00	1.40 (0.54-3.70)	1.23 (0.38-3.99)	0.68
Snoring, n cases/N	17/73	11/46	12/40	
Model 1	1.00	1.11 (0.44-2.78)	1.34 (0.54-3.29)	0.54
Model 2	1.00	1.24 (0.40-3.84)	1.16 (0.37-3.61)	0.76
Model 3	1.00	1.60 (0.48-5.31)	1.19 (0.32-4.41)	0.68
Poor general sleep quality, n cases/N	16/103	18/86	23/78	
Model 1	1.00	1.34 (0.62-2.88)	2.45 (1.14-5.30)	0.02
Model 2	1.00	1.39 (0.58-3.30)	2.70 (1.09-6.72)	0.03
Model 3	1.00	1.53 (0.61-3.80)	2.06 (0.75-5.69)	0.16
Daytime sleepiness[b], n cases/N	25/129	15/107	13/100	
Model 1	1.00	0.68 (0.32-1.42)	0.69 (0.32-1.50)	0.30
Model 2	1.00	0.49 (0.21-1.14)	0.43 (0.17-1.11)	0.06
Model 3	1.00	0.46 (0.19-1.11)	0.35 (0.12-1.00)	0.04

[a] Basal impaired mobility, impaired agility, Katz index≤5 or Lawton-Brody index≥1.

[b] >10 points in the Epworth Sleepiness Scale. Analyses based on 1,240 participants who completed the questionnaire

Model 1: logistic regression model adjusted for age, sex and educational level (≤ primary, secondary, university).

Model 2: logistic regression model additionally adjusted for physical activity (METs-h/wk, tertiles), smoking (never smoker, former smoker, current smoker), alcohol (g/d, tertiles), caffeine (mg/d, tertiles), BMI (<25, 25-29.9, ≥30 kg/m2), diet quality index (MEDAS score, tertiles), energy intake (kcal/d, tertiles) and baseline and incident morbidity, including diabetes, cancer, cardiovascular and musculoskeletal diseases.

Model 3: logistic regression additionally adjusted for sodium (g/d, tertiles), cholesterol (g/d, tertiles) and saturated fatty acids intakes (g/d, tertiles).

© 2018. Lana A et al. Published online at http://www.aginganddisease.org/EN/10.14336/AD.2018.0503

Supplementary Table 4. Odds ratios (95% confidence interval) for the association between meat consumption and both change in sleep duration and number of indicators of poor sleep quality during a 2.8 years follow-up of older adults with some physical impairment[a] (n= 428).

	**Tertile 1 (<87 g/d)**	**Tertile 2**	**Tertile 3 (≥128 g/d)**	**p for trend**	**Continuous per 100 g/d increment**
0 endpoint[b], n	26	17	11		
Reference category	1.00	1.00	1.00		
1 endpoint, n	116	90	75		
Model 1	1.00	1.17 (0.55-2.49)	1.36 (0.59-3.12)	0.45	1.10 (0.59-2.05)
Model 2	1.00	0.98 (0.42-2.23)	1.44 (0.56-3.71)	0.56	1.08 (0.54-2.16)
Model 3	1.00	0.98 (0.41-2.34)	1.26 (0.43-3.66)	0.72	0.98 (0.45-2.15)
2 endpoints, n	23	32	38		
Model 1	1.00	1.78 (0.71-4.45)	4.09 (1.56-10.80)	0.004	2.14 (1.06-4.33)
Model 2	1.00	1.52 (0.56-4.14)	3.89 (1.29-11.75)	0.02	1.81 (0.85-4.16)
Model 3	1.00	1.72 (0.60-4.94)	4.46 (1.27-15.67)	0.02	2.05 (0.85-5.06)

[a] Basal impaired mobility, impaired agility, Katz index≤5 or Lawton-Brody index≥1.
[b] Endpoints are: large changes (increase or decrease) in sleep duration (≥2 hours) and ≥2 indicators of poor sleep quality.
Model 1: multinomial logistic regression adjusted for sex, age (<70, 70-75, ≥75 y), educational level (≤ primary, secondary, university), sleep duration (<7, 7-9, >9 h) in 2012 and indicators of poor sleep in 2012 (≤1, 2-3, ≥4).
Model 2: multinomial logistic regression additionally adjusted for physical activity (METs-h/wk, tertiles), smoking (never smoker, former smoker, current smoker), alcohol (g/d, tertiles), caffeine (mg/d, tertiles), BMI (<25, 25-29.9, ≥30 kg/m^2), diet quality index (MEDAS score, tertiles), energy intake (kcal/d, tertiles) and baseline and incident morbidity, including diabetes, cancer, cardiovascular and musculoskeletal diseases.
Model 3: multinomial logistic regression additionally adjusted for for sodium (g/d, tertiles), cholesterol (g/d, tertiles) and saturated fatty acids intakes (g/d, tertiles).

© 2018. Lana A et al. Published online at http://www.aginganddisease.org/EN/10.14336/AD.2018.0503
